# Individual Variability in Response to Social Stress in Dairy Heifers

**DOI:** 10.3390/ani10081440

**Published:** 2020-08-18

**Authors:** Emeline Nogues, Benjamin Lecorps, Daniel M. Weary, Marina A. G. von Keyserlingk

**Affiliations:** Animal Welfare Program, Faculty of Land and Food Systems, University of British Columbia, Vancouver, BC V6T 1Z4, Canada; enogues@mail.ubc.ca (E.N.); blecorps@mail.ubc.ca (B.L.); danweary@mail.ubc.ca (D.M.W.)

**Keywords:** welfare, social behavior, coping strategy

## Abstract

**Simple Summary:**

Mixing unfamiliar animals (or regrouping) is a common practice on dairy farms. This disruption in the social organization typically results in increased agonistic interactions and changes in time feeding and resting. Our objective was to study if different individuals subjected to a similar regrouping event would display different levels of engagement in social interactions and avoidance of social contact, and if these differences were associated with other behavioral changes. A total of 30 heifers were regrouped. Agonistic behaviors initiated and received, and feeding, resting and standing time and synchronization were recorded. Agonistic interactions were most frequent in the first 6 h after regrouping, with most taking place in the alleyways. Some individuals showed higher levels of engagement in these interactions, and others seemed to avoid aggressive interactions, suggesting different strategies were used to cope with the social stress of regrouping. Heifers that showed a more engaged strategy spent more time feeding. Those that showed higher avoidance spent less time feeding, less time resting and were less synchronized with others in their feeding behavior. We conclude that dairy heifers display different responses to social stress, and that in the case of regrouping, a more engaged strategy is more successful.

**Abstract:**

Regrouping is associated with increased aggression, and disruption of time-budgets. Individuals vary in how well they cope with social stress. Our objective was to describe individual differences in agonistic behavior in dairy heifers after regrouping, and determine how time-budget and behavioral synchronization were affected by these coping strategies. A total of 30 heifers were individually regrouped at 5-months of age into stable groups of 12 unfamiliar animals. For 24 h, agonistic behaviors initiated and received by the regrouped heifer were continuously recorded, and standing, resting and feeding time and synchronization were sampled every 5 min. Scores of engagement in agonistic interactions and avoidance of interactions were calculated for each regrouped heifer. Linear mixed effects models were used to assess whether these two response types were related, and how variation in these responses related to activity and synchronization. Engaged heifers displayed lower avoidance and spent more time feeding. Avoidant heifers spent less time feeding and resting, and were less synchronized while feeding. We conclude that dairy heifers differ in social coping strategy when regrouped through different levels of engagement and avoidance, and that these differences affected their time-budget and behavioral synchronization.

## 1. Introduction

Dairy cattle are gregarious and motivated for social contact [1,2]. Social relationships are affected by the expression of affiliative and agonistic interactions [3]. Stability within a social group is evidenced by a low frequency of agonistic interactions [4]. When the dominance structure is established, there is some evidence that some individuals in the group develop preferential relationships [5,6]. When competition for key resources is low, cattle also tend to synchronize their feeding and resting behaviors [7].

Social stability is disrupted by regrouping unfamiliar individuals, a known stressor in many species including cattle [8], pigs [9] and rats [10]. Regrouping in cattle results in declines in affiliative interactions, such as social grooming, and increases in agonistic interactions [11]. These agonistic interactions include agonistic behaviors such as displacements or fights, and behaviors aimed at avoiding physical aggressions. The majority of studies on regrouping have focused on agonistic behaviors expressed in the context of competition for resources such as feed [12], but agonistic behaviors also occur elsewhere in the pen [13].

Regrouping is a common practice on dairy farms. Newly weaned calves are often regrouped, either because they were previously individually housed, or because groups are comingled post weaning. Eight-week-old calves have been shown to engage in displacement behavior at the feedbunk after regrouping [14], suggesting that this practice can cause conflicts even in younger animals.

The social behavior of farm animals has been argued to be flexible, adapting to the current social and physical environment [15]. Additionally, individuals vary in how well they cope with stress [16]. Previous work on dairy cattle has investigated individual differences in response to stressful stimuli [17,18,19], but responses to social stressors have focused almost exclusively on competition for resources [20,21]. To our knowledge, no studies have explored individual differences in agonistic behavior at regrouping, and whether different behavioral patterns could be identified.

The first aim of this study was to describe the agonistic behaviors initiated and received by dairy heifers when individually regrouped into a pen of unfamiliar conspecifics. We predicted that agonistic interactions would be common shortly after regrouping, and that most would be related to accessing feed. Consistent with Mendl and Deag (1995) [22], we expected that individuals would display different responses when placed in a new and competitive social environment; some were expected to engage in social competition (i.e., displacing other individuals and fighting more often), and others were expected to avoid physical interactions.

Regrouping decreases time spent feeding and resting in dairy cattle and in goats [23,24], and reduces behavioral synchronization in pigs [25]. Under stable conditions, differences in dominance and sociability relate to variability in the feeding behavior of ruminants [26]. In a competitive context, dairy cattle [21] and beef cattle [27] can either actively engage in competition or reduce their feeding synchronization. However, to our knowledge no study has explored whether differences in the agonistic behaviors displayed by dairy heifers after regrouping could explain variations in other changes such as reduced feeding and resting times, or reduced behavioral synchronization.

Our second aim was to explore whether individual differences in engagement and avoidance-based strategies were associated with some benefits in terms of time-budgets, access to resources (i.e., feed), and behavioral synchronization with the new group. We predicted that animals engaging more would be more successful (i.e., spend more time feeding and resting); whereas, animals displaying avoidance would have a reduced access to feed and lower synchronization.

## 2. Materials and Methods

This study was approved by The University of British Columbia’s Animal Care Committee (#A18-0174) and conducted at the UBC Dairy Education and Research Centre in Agassiz, BC, Canada (a herd of 500 animals, of which 250 are lactating cows). We enrolled 33 Holstein heifers, distributed in four stable groups (3 groups of 8 heifers and 1 group of 9 heifers); 3 heifers were excluded a posteriori, due to external disturbances in the barn at the time of regrouping. All individuals were disbudded during the pre-weaning period. Heifers were housed in a sawdust-bedded pen with a concrete feeding alley (providing approximately 7 m^2^ per heifer); new bedding was added weekly. Heifers were provided ad libitum access to water, grain (Heifer starter; Hi-Pro Medicated; Chilliwack, BC, Canada) and hay (mix of long stemmed tall fescue and orchard grass); each type of feed was available in a separate feeding trough that could only be accessed by one animal at a time (RIC; Hokofarm Group B.V., Marknesse, The Netherlands).

Every week, we regrouped two heifers into two separate host groups at 08.00 h, immediately before fresh feed delivery; heifers remained in the regrouped pen for 72 h before they were returned to their initial group. Across all four initial groups, the heifers were on average 149 ± 16 days old (mean ± SD) at the time of regrouping. The host groups (n = 8) in the regrouping pens were composed of 12 unfamiliar heifers, on average 46 ± 7 days older (mean ± SD) than the regrouped animals. Focal heifers were regrouped into a free-stall pen (providing approximately 4.8 m^2^ per heifer) with concrete alleys, and fitted with 13 deep-bedded stalls (2 × 0.9 m each; bedded with sand). Heifers were provided a total mixed ration (TMR; mixture of grain, straw, alfalfa hay, grass and corn silage) accessible through 15 self-lock head gates (35 cm center-to-center) fed once daily at 09.00 h. Once all the heifers in the initial group had been regrouped the next group was started. At the beginning of each of the four initial groups, the host heifers in the regrouping pens were replaced and allowed to habituate for two weeks.

Two cameras (WV-CW504SP, Panasonic, Osaka, Japan) were placed 8 m above each of the regrouping pens. All behavioral measures were obtained through video. Animals were identified using their unique coat patterns. As observers were required to follow the regrouped heifer, they could not be blind to treatment (i.e., regrouped or host).

Agonistic dyadic interactions involving the regrouped individual were continuously recorded during the first 24 h of regrouping. These behaviors included displacements, threats, avoidances and fights; following the definitions given by Gibbons et al. [28]. All agonistic behaviors started as one individual approaching another, one of which was the regrouped heifer. If the approaching individual pushed away the other using head-to-head, head-to-neck or head-to-flank contact, the interaction was recorded as a displacement. Displacements were categorized separately depending upon the role of the regrouped heifer (i.e., if she was the one pushing or receiving the push), and where the displacement occurred: alleyways, feedbunk (head was through the headlocks) or stall (standing with at least the two forelegs in the stall, or lying). If following the approach of one heifer to another, both engaged in a head-to-head contact lasting at least 3 s, the interaction was recorded as a fight. If the approach resulted in one animal moving away thereby avoiding physical contact, the interaction was recorded as either a threat or an avoidance. In the case of a threat the regrouped heifer approached a host animal, including positioning her forehead in the direction of the host; this may have also included a swing of the head. In the case of an avoidance the host heifer approached the regrouped heifer and the latter retreated; no distinction was made based on the approaching heifer’s behavior (i.e., position of the forehead or head swing was not required).

Activity was categorized into standing, resting and feeding. The activity of each heifer (regrouped and hosts) was recorded using instantaneous scan sampling every 5 min for 24 h, beginning when the regrouped heifer first entered into the regrouping pen. Synchronization for each activity (i.e., standing, resting and feeding) was estimated by counting the number of host heifers involved in the same activity as the regrouped individual at each scan and averaging over the 24 h.

R version 3.3.2 was used for all statistical analyses (raw data and R script are available in the Supplementary Materials). Inter-observer reliability was evaluated using the intraclass correlation coefficient (ICC, package ICC) applied to a random selection of 12 h of video, for which standing, resting and feeding behaviors of the host group were sampled by four observers. Observers showed excellent agreement (ICC = 0.99, CI: 0.98–0.99). Two observers recorded the agonistic interactions and showed good agreement for all behaviors (ICC ranging from 0.55 to 0.88; see Supplementary Materials Table S1 for detail by behavior), except for threats initiated by the regrouped heifer (ICC = 0.23); given the poor agreement for this latter measure it was excluded from further analysis.

Animals displacing (independently of the location) and fighting more frequently were considered as more engaged (see Equation (1)); whereas, animals avoiding interactions more frequently were considered as being more avoidant.

(1) Engagement=Displacements initiated in all three locations+Fights

All linear mixed-effects models were built with the lme() function (package nlme) with host group as a random effect, and *F* values and *p* values of the Wald tests were extracted using the anova.lme() function (package nlme). The first model built had avoidance as a response variable and engagement as a fixed effect. Secondly, four models were built with engagement as a fixed effect and either time spent feeding or resting, or synchronization while feeding or resting as response variable. Similarly, four models were built with avoidance as a fixed effect and either time spent feeding or resting, or synchronization while feeding or resting as response variable. Time spent standing and synchronization while standing were not tested to limit the number of inferential tests. Model assumptions were checked using graphical analysis of their residuals. One heifer was an outlier for avoidances, as identified graphically on a boxplot. To avoid having to remove her from the analysis, her true value for avoidances was replaced by the second highest value + 1. Exploratory analyses were conducted including the true value, the capped value or excluding this heifer, and we identified no differences in significance. In all the models including engagement as an independent variable, engagement was log transformed to linearize the relationship. The distribution of the residuals from the model with time spent feeding as a function of engagement was positively skewed, to satisfy model assumption, time spent feeding was also log transformed. The distribution of the residuals from the model with synchronization while feeding as a function of engagement was negatively skewed; to satisfy model assumption, synchronization while feeding was squared.

## 3. Results

**Figure 1:**
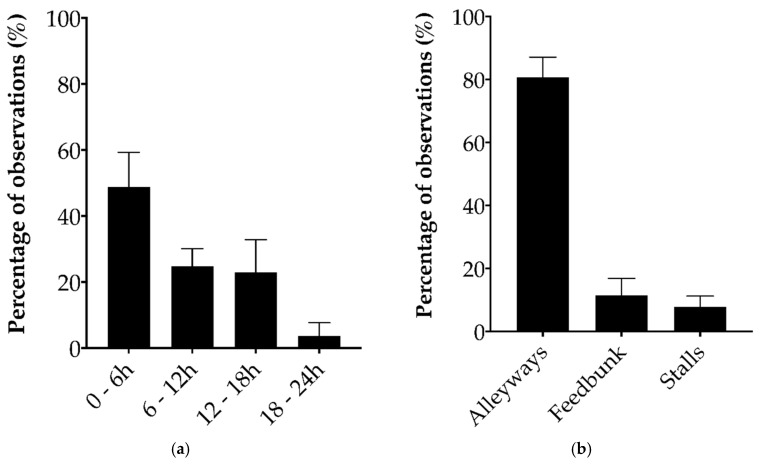
Percentage (mean ± SD) of agonistic behaviors initiated and received by dairy heifers during the first 24 h of a regrouping event (n = 30) in relation to: (**a**) four 6-h periods, (**b**) the location within the pen where the agonistic behavior was observed.

Agonistic interactions occurred most frequently within 6 h of regrouping, and in the alleyways (Figure 1). Each agonistic behavior recorded followed the same temporal distribution (detailed in Table S2). Displacements and avoidances were the most frequent behaviors (respectively 45.7 ± 11.7% and 34.9 ± 17.9%; mean ± SD). Regrouped heifers received between 51 to 554 agonistic interactions over the 24-h test period, and initiated between four to 246 interactions over the same period. Out of the 30 regrouped heifers, only three initiated more agonistic behaviors than they received.

**Figure 2:**
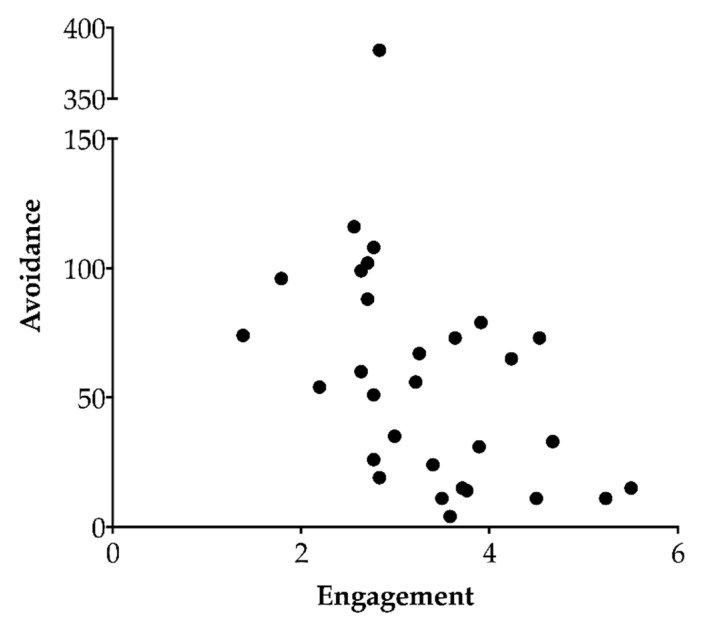
The relationship between avoidance and engagement levels (log transformed) of dairy heifers (n = 30) regrouped with unfamiliar conspecifics. Agonistic behaviors were recorded continuously during the first 24 h after heifers were introduced into a new group. More engaged individuals displayed less avoidance (Wald test on a linear mixed-effects model with host group as a random effect; *p* = 0.02). The extreme point for avoidance was capped in the model at the second highest value + 1.

The heifers in this study varied in levels of engagement and avoidance, and these two behavioral patterns were negatively related (*F*_1,21_ = 6.34, β = −12.91, *p* = 0.02; Figure 2).

**Figure 3:**
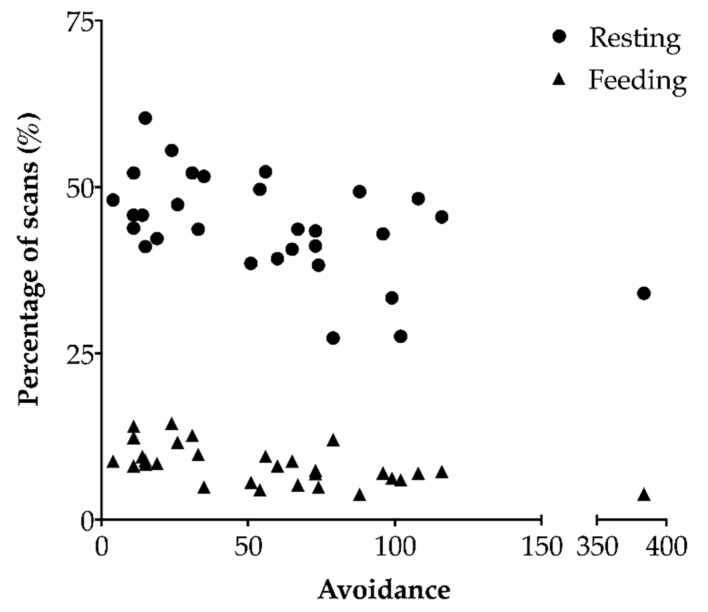
The relationship between each regrouped heifer’s avoidance level (n = 30) and the percentage of scans they were recorded feeding or resting (samples were taken every five minutes). Agonistic behaviors were recorded continuously during the first 24 h after heifers were introduced into a new group. More avoidant heifers spent less time feeding (Wald test on a linear mixed-effects model with host group as a random effect; *p* = 0.0014) and less time resting (*p* = 0.01). No relation was found between avoidance and time spent resting. The extreme point for avoidance was capped in the model at the second highest value + 1.

More engaged heifers spent more time feeding (*F*_1,21_ = 10.20, β = 0.20, *p* = 0.0044), but there was no relation between engagement and time spent resting (*F*_1,21_ = 0.068, β = 0.39, *p* = 0.80). More avoidant heifers spent less time feeding (*F*_1,21_ = 13.51, β = −0.047, *p* = 0.0014; Figure 3), and less time resting (*F*_1,21_ = 8.017, β = −0.11, *p* = 0.01).

**Figure 4:**
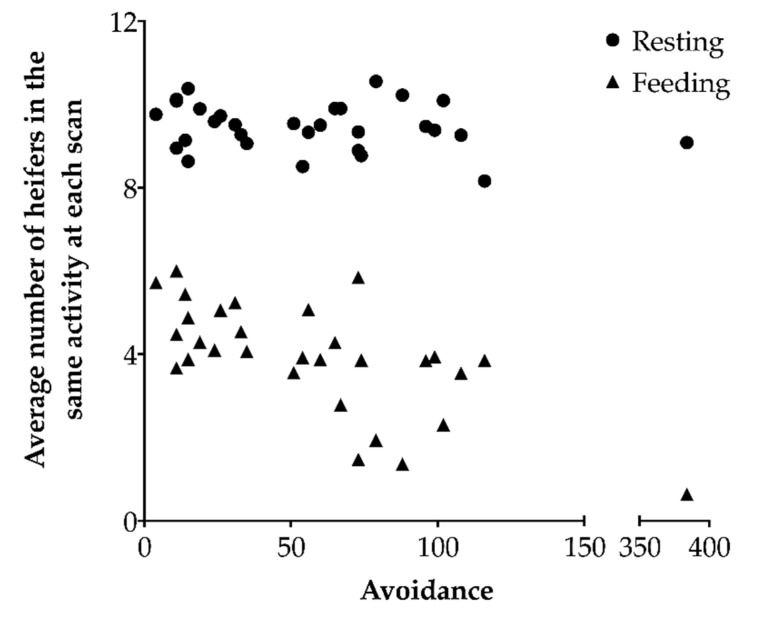
The relationship between each regrouped heifer’s avoidance (n = 30) and behavioral synchronization for feeding or resting. Synchronization was assessed by counting the number of individuals involved in the same activity as the regrouped heifer at each scan, and averaging over the number of scans the heifer was recorded as either feeding or resting. Agonistic behaviors were recorded continuously during the first 24 h after heifers were introduced into a new group. More avoidant heifers were less synchronized while feeding (Wald test on a linear mixed-effects model with host group as a random effect; *p* = 0.013), but no relationship was found with synchronization while resting (*p* = 0.36). The extreme point for avoidance was capped in the model at the second highest value + 1.

More engaged heifers tended to be more synchronized with the other heifers in the pen while resting (*F*_1,21_ = 3.25, β = 0.19, *p* = 0.086), but there was no relation between the level of engagement and synchronization while feeding (*F*_1,21_ = 0.87, β = 1.60, *p* = 0.36). More avoidant heifers were less synchronized with the other heifers in the pen while feeding (*F*_1,21_ = 11.51, β = −0.021, *p* = 0.0027; Figure 4), but there was no relation between the level of avoidance and synchronization while resting (*F*_1,21_ = 0.87, β = −0.0031, *p* = 0.36).

## 4. Discussion

This study describes agonistic behaviors initiated and received by dairy heifers when regrouped into a new social group. We explored whether heifers differed in their agonistic responses to regrouping and how these differences related to other behaviors (i.e., feeding and resting time and synchronization).

Our results agree with previous work on regrouping indicating that agonistic behaviors are frequent after regrouping [23], particularly within the first 6 h. Competition for resources can trigger high levels of agonistic interactions in cattle [29]. As heifers in this study were regrouped at 08.00 h, shortly before fresh feed delivery, the increase in aggressive interactions due to the regrouping cannot be disentangled from the effect of fresh feed delivery. Despite the possibility of a circadian pattern affecting agonistic interactions, we do not believe that regrouping further away from the time of fresh feed delivery would reduce aggression, as previous work has shown no reduction in displacements when regrouping occurred outside of the time of fresh feed delivery [30]. Most work to date has focused on competition to access feed or lying spaces after regrouping [31]. However, regrouped animals also use agonistic behaviors to establish their position within the social hierarchy. A study on pigs found that provision or deprivation of food and water did not affect the number of agonistic interactions after regrouping [32]. In the current study, many agonistic interactions took place in the alleyways suggesting that regrouping triggers aggression independent of access to a specific resource. These results suggest that earlier work on regrouping in cattle, focusing primarily on access to feed or lying stalls, may have underestimated agonistic interactions.

Of the agonistic interactions recorded, some of the interactions recorded as ‘fights’ might actually have been expressions of play behavior. However, we suggest that the risk of this mis-categorization was low, given that the regrouped heifers had no previous contact with the host cattle.

In the current study, heifers displayed considerable variability in agonistic behavior following regrouping. Different coping styles, defined as stable behavioral strategies when facing a challenge [33], have been described in mammals in response to stressors. Previous work on social stress in other species showed that rats [33], great tits [34] or pigs [35] with a more proactive coping style typically engage with threats by displaying agonistic behaviors. Our results suggest that some heifers express a more proactive coping strategy, based on being more aggressive (i.e., engaging with the threat), whereas others display a more reactive coping strategy, based on avoidances of physical interactions. Our hypothesis is that the observed variability in behavioral response is an individual characteristic of the regrouped heifer.

Although aggressiveness has been described as a stable individual trait in dairy cattle [28], it remains unclear whether the coping strategy employed by individuals would persist across regrouping events or in other socially stressful situations. In a study in pigs, the individual level of aggression at regrouping was a poor predictor of aggression after the group structure stabilized [36]. The strategies described in this study are consistent with those previously found in beef cattle subjected to increased competition [27]. Social interactions might be related to other personality traits. Highly sociable individuals (i.e., those spending more time in proximity with conspecifics and more stressed by social isolation [19,37]), may be more sensitive to changes in their social environment, but might also be more eager to integrate a new social group. Recent evidence also suggests that individuals’ levels of pessimism may affect social interactions; more pessimistic calves displayed higher social avoidance (i.e., they interacted with only a few group-members) [38]. In this study, we specifically focused on the regrouping event, but future research should investigate the effect of specific personality traits on response to various stressors.

In this study, regrouped heifers were on average a month and a half younger than their hosts. Although we did not record body weight, the regrouped heifers were clearly smaller than the older animals they were regrouped with. Differences in age and body size may have affected social interactions and dominance: younger and smaller cattle are usually lower ranking [39]. The heifer’s rank in the group of origin, and the relative change in dominance status associated with the regrouping event, can also affect the regrouped individual [40]. All heifers in this study were raised under similar social conditions, but previous studies showed that rearing calves in isolation or with their mothers during the milk-feeding period influenced their social behaviors when integrated into new social groups [41]. Future work could explore how previous dominance and other social experience may influence the way animals cope with regrouping, and whether more dominant individuals would engage in more interactions, while it is expected that subordinates would avoid interactions and adapt their time-budget.

Regrouping negatively affects time-budget [23], which can, in turn, disrupt behavioral synchronization. Heifers expressing more avoidance spent less time feeding, less time resting and were also less synchronized in their feeding behavior. These results support the idea that these animals adapted their behaviors to avoid peak feeding times as means of avoiding competition and associated physical aggressions, and most likely fed when the hosts were resting. However, the quality and quantity of the feed declines in the hours after fresh feed delivery [42], so reduced time spent feeding and reduced feeding synchronization may be costly to the regrouped individual. Social stress such as regrouping has been reported to more negatively affect low-ranking dairy cows [39] (i.e., individuals that are already less likely to access key resources, such as fresh feed, under stable conditions [13,43]).

Some heifers would rather engage in aggressive interactions than alter their feeding behavior. Not surprisingly, these individuals also tended to be more synchronized while resting, most likely because having been able to feed, they were more motivated to rest. In the case of individual regrouping, displaying a more engaged social coping strategy appears to be more successful in maintaining access to feed.

## 5. Conclusions

Regrouping of dairy heifers triggers increased agonistic interactions, especially within the first 6 h and taking place primarily in the alleyways. Dairy heifers display individual variability in their response to regrouping, affecting time budgets and behavioral synchronization. Animals with a more engaged coping strategy appeared to be more successful at mitigating the negative effects of regrouping than those using an avoidance-based strategy.

## Supplementary Materials

The following are available online at https://www.mdpi.com/2076-2615/10/8/1440/s1. Table S1: ICC of the two observers of agonistic behaviors, Table S2: Temporal distribution of each agonistic behavior, Supplementary Data 1: Raw data used for statistical analysis, Supplementary File 1: Description for Supplementary Data 1, Supplementary File 2: R script for statistical analysis.
