# Supplementary material for: Individual Variability in Response to Social Stress in Dairy Heifers
Source: Animals (Basel). 2020 Aug 18;10(8):1440. doi: 10.3390/ani10081440 (PMC7459822; doi:10.3390/ani10081440)
Supplement: Supplementary file 1 [file animals-10-01440-s001.zip › Supplementary material/Supplementary_File_1.docx]

*Description for supplementary file* ***Supplementary_Data_1.csv****.*

The file contains the raw data used for statistical analysis. Columns in this table represent the following variables:

**Heifer:** individual animal ID

**Group:** host group ID

**Disp_Feed_I:** no. of displacements initiated by the regrouped heifer at the feedbunk

**Disp_Stall_I:** no. of displacements initiated by the regrouped heifer in the stalls

**Disp_Alley_I:** no. of displacements initiated by the regrouped heifer in the alley

**Disp_Feed_R:** no. of displacements received by the regrouped heifer at the feedbunk

**Disp_Stall_R:** no. of displacements received by the regrouped heifer in the stalls

**Disp_Alley_R:** no. of displacements received by the regrouped heifer in the alley

**Avoidances:** no. of avoidances displayed by the regrouped heifer

**Fights:** no. of fights the regrouped heifer was implicated in

**Pct_Standing:** percentage of scans the regrouped heifer was recorded standing

**Pct_Resting:** percentage of scans the regrouped heifer was recorded resting

**Pct_Feeding:** percentage of scans the regrouped heifer was recorded feeding

**Sync_Standing:** average no. of individuals also standing when the regrouped heifer was recorded standing

**Sync_Resting:** average no. of individuals also resting when the regrouped heifer was recorded resting

**Sync_Feeding:** average no. of individuals also feeding when the regrouped heifer was recorded feeding

The code for generating the results in the manuscript is provided in the ***Supplementary_File_2.R*** file.
