# Supplementary material for: Individual Variability in Response to Social Stress in Dairy Heifers
Source: Animals (Basel). 2020 Aug 18;10(8):1440. doi: 10.3390/ani10081440 (PMC7459822; doi:10.3390/ani10081440)
Supplement: Supplementary file 1 [file animals-10-01440-s001.zip › Supplementary material/Table S1 and Table S2.docx]

**Supplementary material - Tables**

**Table S1:** Reliability of the two observers in scoring the different agonistic behaviours displayed by dairy heifers when regrouped. Reliability was calculated based on the observations of the first 24 h immediately after regrouping for 4 heifers (recordings were divided in 4 periods of 6 h; n=16 observations for each behaviour).

| **Behaviour** | **ICC** | **CI** |
| --- | --- | --- |
| Displacements received in the alleys | 0.88 | 0.69 – 0.95 |
| Displacements received at the feedbunk | 0.66 | 0.27 – 0.86 |
| Displacements received in the stalls | 0.69 | 0.33 – 0.88 |
| Displacements initiated in the alleys | 0.55 | 0.11 – 0.81 |
| Displacements initiated at the feedbunk | 0.79 | 0.50 – 0.92 |
| Displacements initiated in the stalls | 0.72 | 0.38 – 0.89 |
| Avoidances | 0.81 | 0.54 – 0.93 |
| Threats | 0.23 | -0.27 – 0.64 |
| Fights | 0.86 | 0.66 – 0.95 |

**Table S2:** Temporal distribution of each agonistic behaviour averaged over the regrouped heifers (± SD; n = 30). All behaviours follow the same pattern: from most observed in the first 6 hrs following regrouping, and progressively decreasing over the following 18 hrs.

| **Behaviour** | **Period** | **Mean ± SD (%)** |
| --- | --- | --- |
| Displacements received in the alleys | 0 – 6h | 46.78 ± 12.37 |
|  | 6 – 12h | 25.57 ± 8.10 |
|  | 12 – 18h | 23.36 ± 10.68 |
|  | 18 – 24h | 4.29 ± 6.52 |
| Displacements received at the feedbunk | 0 – 6h | 40.03 ± 24.47 |
|  | 6 – 12h | 29.00 ± 21.06 |
|  | 12 – 18h | 24.87 ± 23.78 |
|  | 18 – 24h | 6.10 ± 11.64 |
| Displacements received in the stalls | 0 – 6h | 52.45 ± 28.59 |
|  | 6 – 12h | 20.99 ± 17.04 |
|  | 12 – 18h | 24.13 ± 25.78 |
|  | 18 – 24h | 2.43 ± 5.57 |
| Displacements initiated in the alleys | 0 – 6h | 48.70 ± 31.87 |
|  | 6 – 12h | 26.53 ± 27.56 |
|  | 12 – 18h | 18.31 ± 20.72 |
|  | 18 – 24h | 6.46 ± 19.07 |
| Displacements initiated at the feedbunk | 0 – 6h | 47.98 ± 29.29 |
|  | 6 – 12h | 26.79 ± 24.54 |
|  | 12 – 18h | 22.03 ± 24.48 |
|  | 18 – 24h | 3.20 ± 7.02 |
| Displacements initiated in the stalls | 0 – 6h | 49.94 ± 43.32 |
|  | 6 – 12h | 19.97 ± 29.65 |
|  | 12 – 18h | 20.10 ± 30.74 |
|  | 18 – 24h | 10.00 ± 28.87 |
| Avoidances | 0 – 6h | 44.94 ± 16.21 |
|  | 6 – 12h | 22.78 ± 9.54 |
|  | 12 – 18h | 29.07 ± 17.92 |
|  | 18 – 24h | 3.22 ± 4.56 |
| Fights | 0 – 6h | 63.41 ± 27.75 |
|  | 6 – 12h | 21.65 ± 23.41 |
|  | 12 – 18h | 9.78 ± 11.50 |
|  | 18 – 24h | 5.16 ± 12.72 |
