# Supplementary figures and images for: Individual Variability in Response to Social Stress in Dairy Heifers
Source: Animals (Basel). 2020 Aug 18;10(8):1440. doi: 10.3390/ani10081440 (PMC7459822; doi:10.3390/ani10081440)

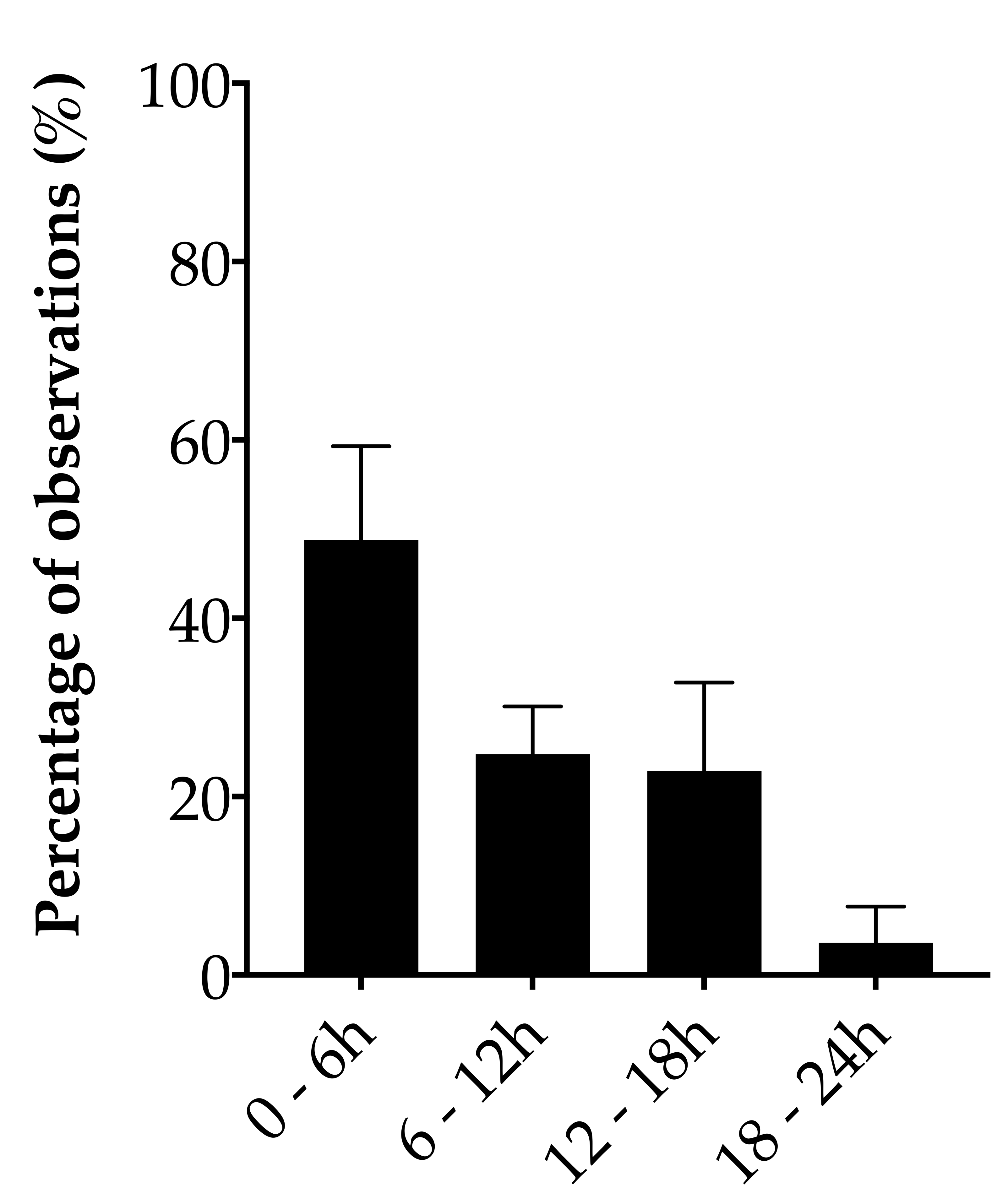

Supplement: Supplementary file 1 [file animals-10-01440-s001.zip › Supplementary material/Figures/Figure1a.tiff]

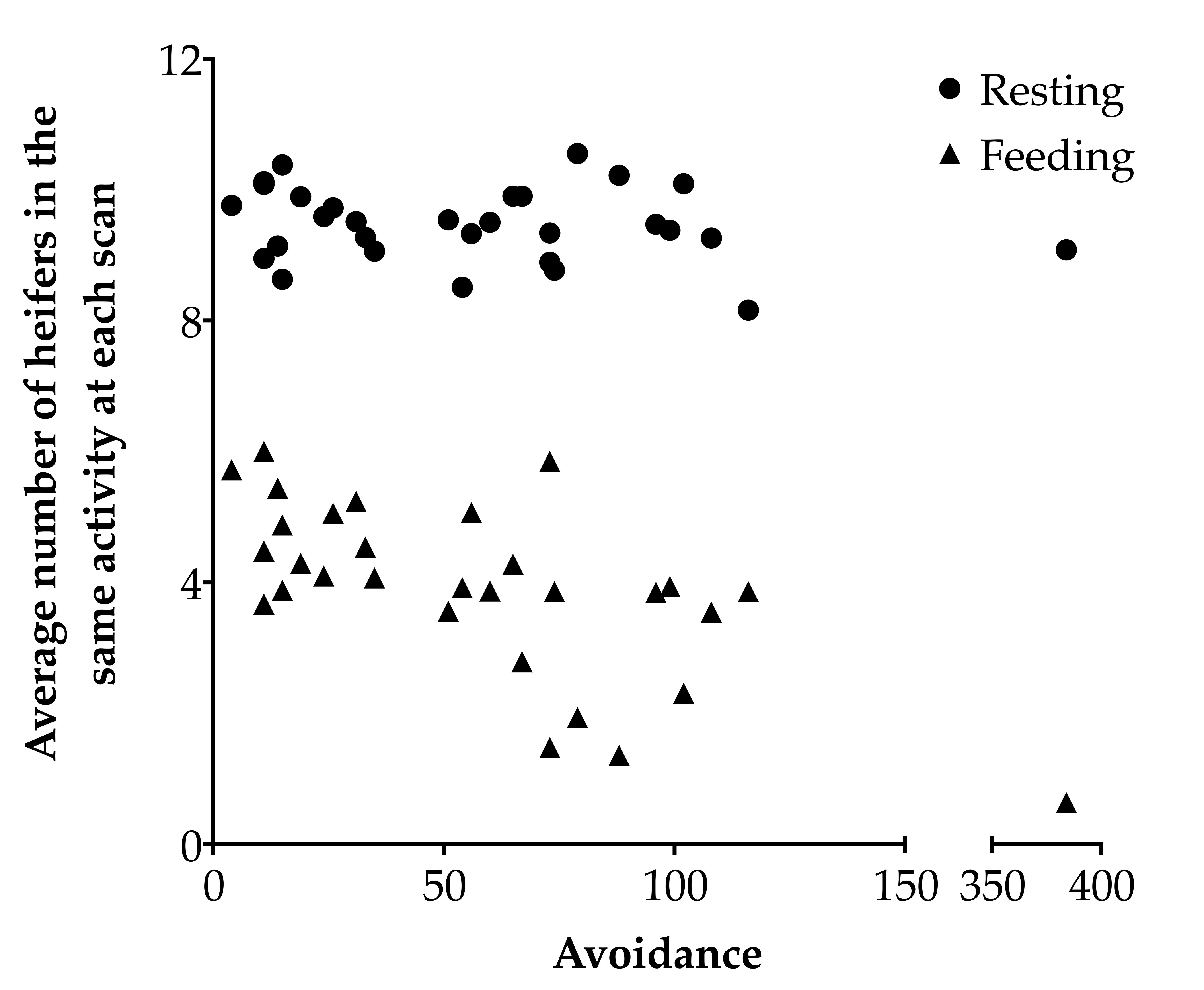

Supplement: Supplementary file 1 [file animals-10-01440-s001.zip › Supplementary material/Figures/Figure4.tiff]

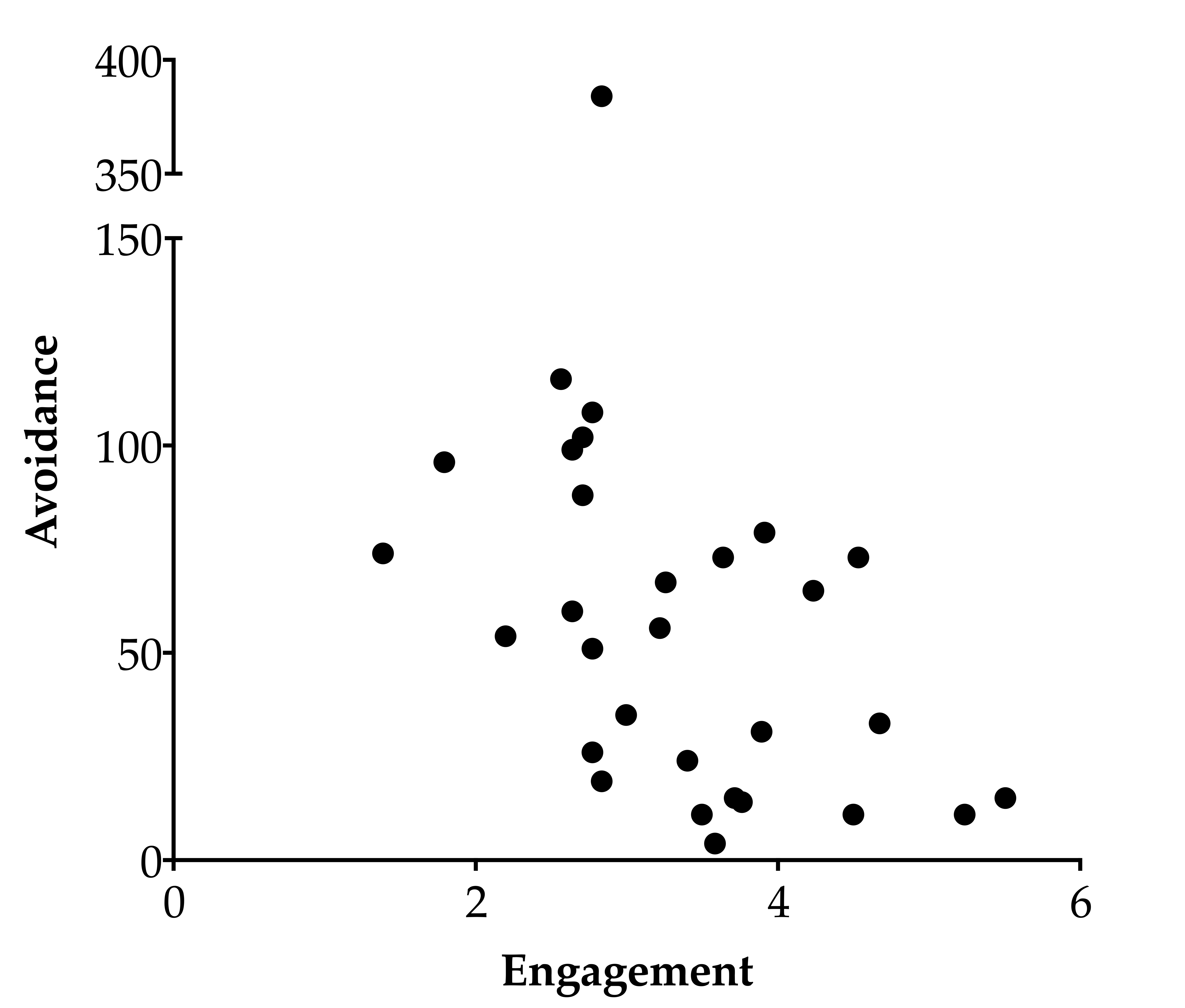

Supplement: Supplementary file 1 [file animals-10-01440-s001.zip › Supplementary material/Figures/Figure2.tiff]

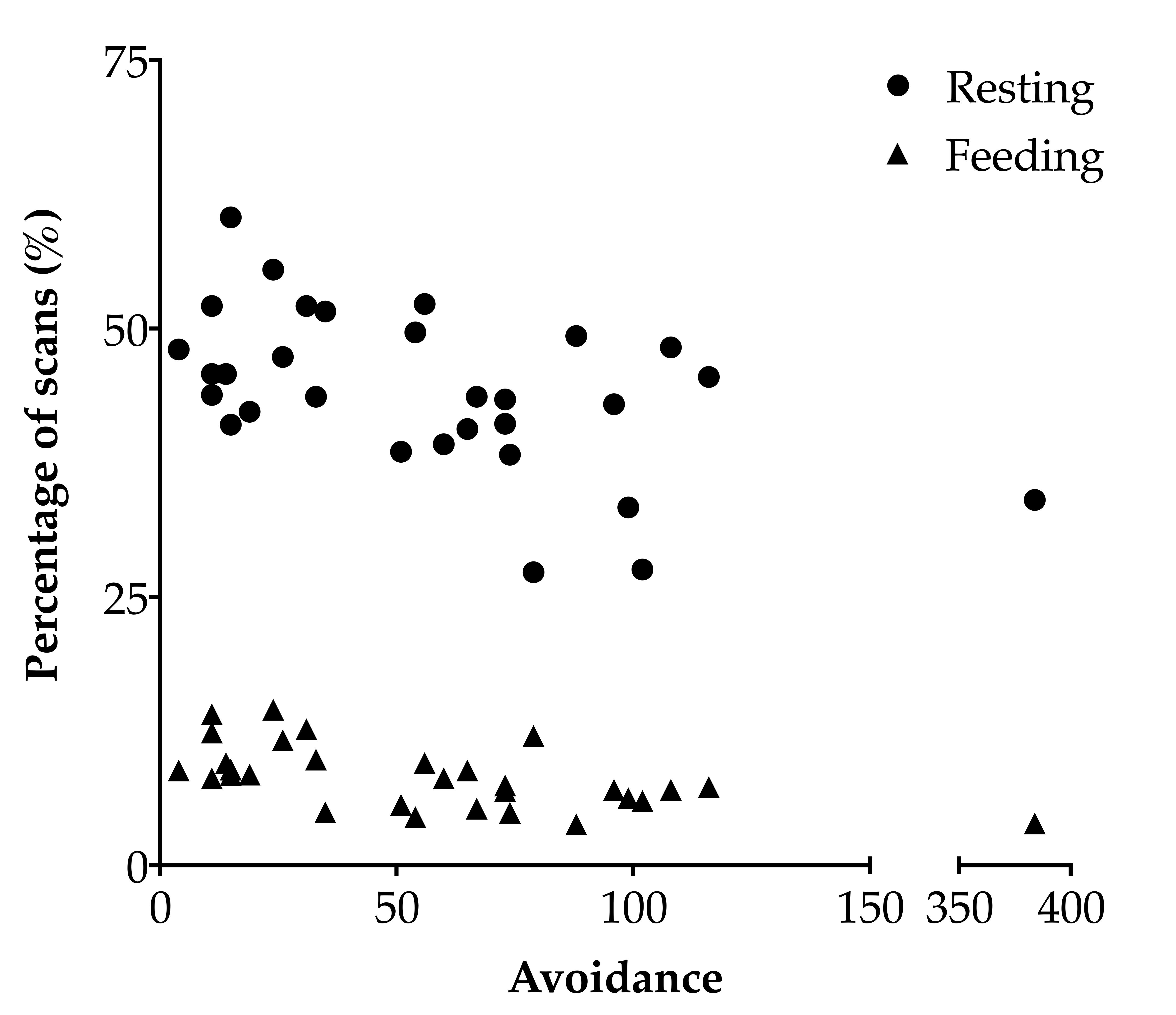

Supplement: Supplementary file 1 [file animals-10-01440-s001.zip › Supplementary material/Figures/Figure3.tiff]

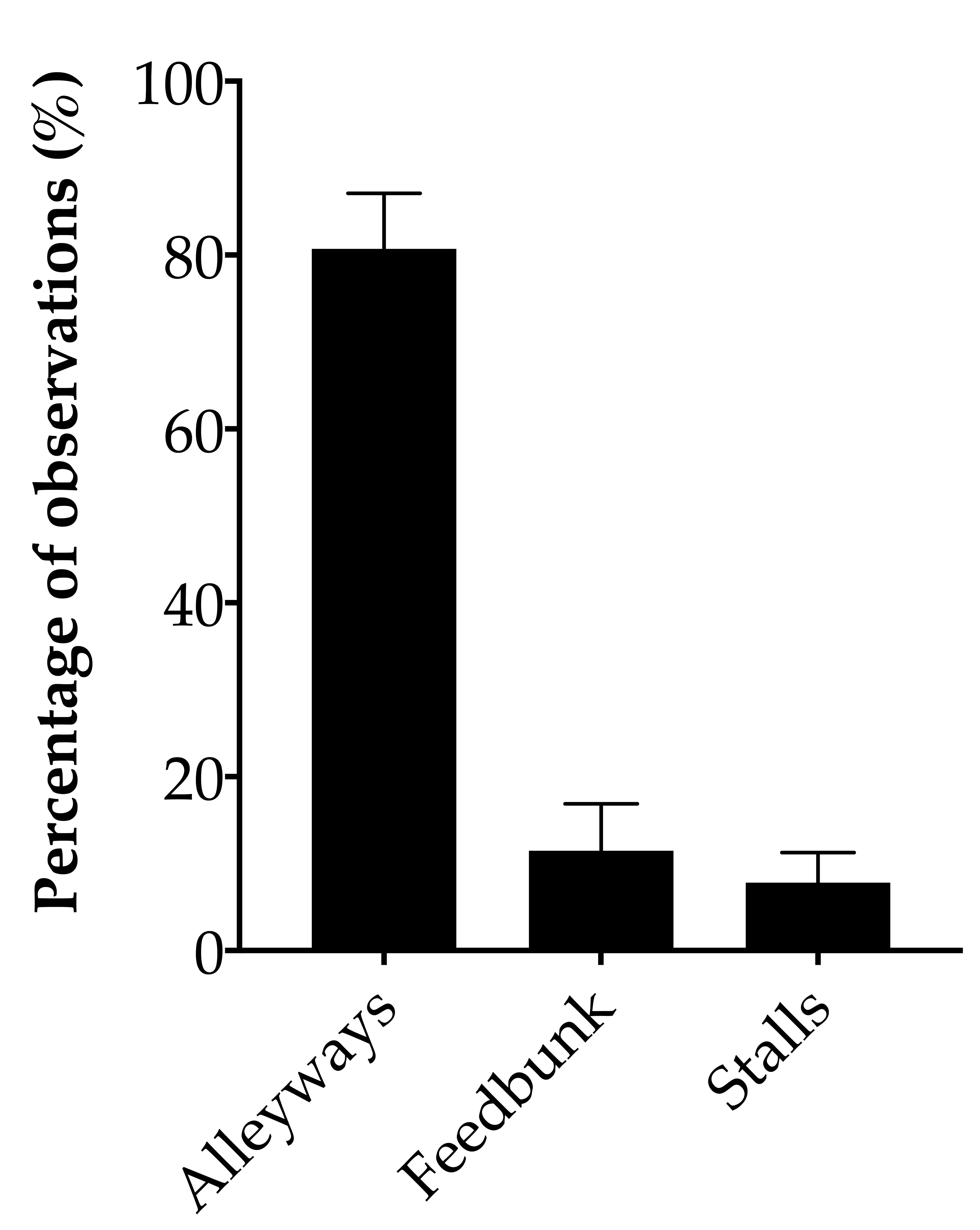

Supplement: Supplementary file 1 [file animals-10-01440-s001.zip › Supplementary material/Figures/Figure1b.tiff]
